# Terrestrial isopods (Oniscidea) of the White Carpathians (Czech Republic and Slovakia)

**DOI:** 10.3897/zookeys.801.24133

**Published:** 2018-12-03

**Authors:** Karel Tajovský, Jana Štrichelová, Ivan H. Tuf

**Affiliations:** 1 Institute of Soil Biology, Biology Centre, Czech Academy of Sciences, České Budějovice, Czech Republic Institute of Soil Biology, Biology Centre, Czech Academy of Sciences České Budějovice Czech Republic; 2 Department of Ecology and Environmental Sciences, Palacky University Olomouc, Czech Republic Palacky University Olomouc Olomouc Czech Republic

**Keywords:** Czech Republic, Isopoda, Oniscidea, Slovakia, terrestrial isopods, Western Carpathians, White Carpathians

## Abstract

This paper summarizes data regarding the terrestrial isopods of the White Carpathians range in the Western Outer Carpathians based on field research undertaken during the past several decades in natural meadow pasture and forest localities. Using a combination of four collection methods 19 species belonging to nine families were recorded. The most common representatives were *ProtracheoniscuspolitusTrachelipusrathkii* and *Ligidiumhypnorum*. In general the biodiversity of isopod communities in the studied area was considerable with half of the localities explored inhabited by six to ten species. The composition of the isopod assemblages was determined by the character of the biotope and its geographical location. Forest habitats were considerably richer in species than the meadow and pasture ecosystems. Some xerotermic localities in the Slovak part of the area were inhabited by *Trachelipusnodulosus* and *Orthometoponplanum* thermophilic species typically associated with warmer parts of Europe. Two relic species (*Hyloniscusmariae* and *Ligidiumgermanicum*) were confirmed for this area. Except for only one finding of *Porcellioscaber* no other evidently introduced or synanthropic species were recorded. Based on the data analyzed the high nature conservancy value of the given area is emphasised.

## Introduction

**Figure 1.**
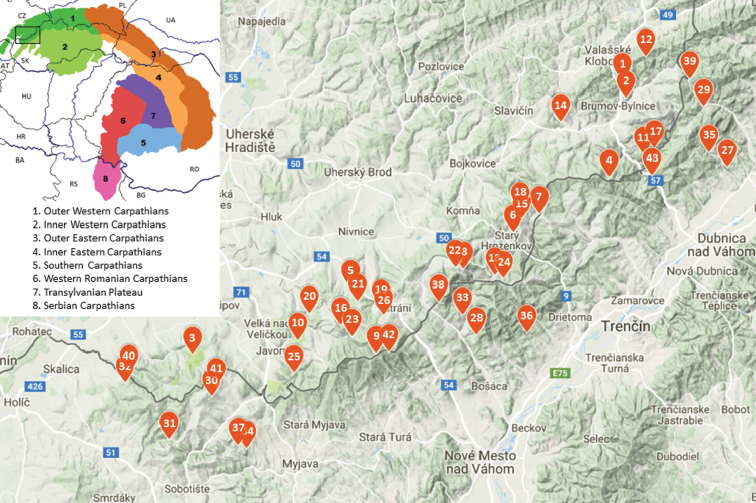
The zonation of Carpathians, with enlarged inset part of position of studied localities in CZ/SK White Carpathians. Source of the map of the Carpathian zones: https://commons.wikipedia.org/wiki/File:Mapcarpat2.png accessed 24.7.2018; the map with position of studied localities according to Google Maps.

The Carpathian range measures approximately 1,500 km and covers ca. 203,000 km^2^. The entire Carpathian chain is usually divided into three major parts: the Western Carpathians (Austria, the Czech Republic, southwestern Poland, Slovakia and Hungary), the Eastern Carpathians (southeastern Poland, eastern Slovakia, Ukraine and Romania), and the Southern Carpathians (Romania and Serbia). The Western Carpathians comprise ca. 70,000 km^2^ and are divided into the four geological zones: 1) an outer flysch zone; 2) a zone with isolated limestone outcrops; 3) a central zone with transformed and underground igneous rocks; 4) a zone with limestone sediments as well as an inner zone with overground igneous rocks. In the Czech Republic, only a part of the Outer Western Carpathians (Figure 1) is situated in the south-eastern Moravia, constituted from west to east by the South-Moravian Carpathians, Central Moravian Carpathians, Slovak-Moravian Carpathians, West-Beskidian Piedmont and, in part, the Western Beskids. Due to its geological and geographic development, this area is (with the exception of other parts of the Central European Hercynian Mountains) distinctive in its vegetation as well as faunal composition.

Research regarding isopod fauna in the Czech part of the Carpathians was initiated by Frankenberger (1941, 1942, 1944, 1954, 1959). He subsequently published data about several species from the Pálava Hills (South-Moravian Carpathians), Chřiby Hills (Central Moravian Carpathians), Vsetínské vrchy Hills, the surroundings of the town of Vizovice, the White Carpathians (all within the Slovak-Moravian Carpathians) and the Moravskoslezské Beskydy Mountains (Western Beskids). One of the most interesting findings was the record of *Hyloniscusmariae*, on the Solánec peak (located in the Vsetínské vrchy Hills), a Carpathian species that at the time was known only in Slovakia. Later, Frankenberger (1944) identified *Trachelipusdifficilis* in the Beskydy Mountains (mentioned as *T.waechtleri*). Flasarová (1958) investigated isopod fauna in the Vsetínské vrchy Hills and the Chřiby Hills and announced 10 species, including the species *Hyloniscusmariae*. Spitzer et al. (2007), who investigated soil fauna in fir-beech forests of the Vsetínske vrchy Hills through the sole use of pitfall trapping, found four isopod species.

The White Carpathians are geographically located along the border between the Czech Republic and Slovakia, and constitute one of the westernmost parts of the entire mountain range, with a relatively high altitude that reaches above 900 m a.s.l. in the peaks. A large part of the territory of the White Carpathians on both the Czech and Slovak sides is designated a Protected Landscape Area (PLA).

In Slovakia, the isopod fauna of the White Carpathians has yet to be studied. Only in the 1990s, selected localities of importance to conservation in the Slovak part of the White Carpathians, were sampled for terrestrial isopods by †Pavel Deván. These were submitted to the first author of this contribution for study, but have not been elaborated. The Little Carpathians, which lie along the southern part of the White Carpathians but are orographically linked to the Inner Carpathians, were surveyed by Flasarová (1980, 1986) and Flasar and Flasarová (1989) via intensive sampling at more than 50 localities. Flasarová recorded a total of 27 species from both natural and synanthropic habitats, and reported noteworthly species *Hyloniscustranssilvanicus* (Verhoeff, 1901) at a single locality in Slovakia, as well as *Armadillidiumzenckeri* Brandt, 1833. Moreover, Kuracina and Kabátová (2005) investigated the locality Devínska Kobyla, which also belongs to the Little Carpathians. Unfortunately, their data regarding 12 species are rather dubious owing to their apparently inaccurate determination and the researchers’ inability to verify missing material (A. Mock pers. comm.). Other research (Tuf and Tufová 2005) primarily targeted isopod communities in oak-hornbeam forests in this area. Štrichelová and Tuf (2012) recorded 10 species in the city of Bratislava and its surrounding whilst investigating localities belonging to the Little Carpathians (except for two urban ones). To date, 30 species have been recorded in the Little Carpathians.

The Czech part of the White Carpathians PLA was explored for terrestrial isopods by Tajovský (2008). He studied meadow and grassland habitats, focusing on the effects of grazing on soil biodiversity. He recorded 14 species, and demonstrated that intensive grazing had a negative impact on the abundance and species richness of soil fauna. Recently, the last author and his students explored predominantly forest localities in the White Carpathians within a series of faunistic inventories of soil fauna in protected areas, but this work has yet to be published.

In this paper, we summarize data from a wide spectrum of biotopes in both the Czech and Slovak parts of the White Carpathians, based on the published records and elaboration of all available material regarding terrestrial isopods. Our results provide basic information about the isopod fauna of this part of the Western Carpathian, facilitating comparison with other areas of the Carpathian mountain range as a whole.

## Materials and methods

The target area, which is protected as the bilateral White Carpathians PLA in both the Czech Republic and Slovakia, is situated along the border of these countries. The Czech part is 70 km long, with a northeast-southwest orientation and an altitude ranging from 175 to 970 m a.s.l. The PLA was established in 1980 on a territory measuring 747 km^2^. Typical of the southern part is a vast complex of species-rich calcareous meadows with dispersed, solitary trees. The landscape in the central part of PLA was created between the 17^th^ and 18^th^ centuries during the Wallachian colonization. It is characterized by scattered houses, alternating forest and non-forest areas, with a mozaic of wetlands, small forests, shrubs and patches of grassland. The northeastern part is situated at a higher altitude and is mainly covered by old-growth beech forests (Mackovčin et al. 2002).

In the present contribution, we surveyed terrestrial isopod fauna in 26 localities representing different natural habitats of the White Carpathians in the Czech Republic between 2002 and 2009, as well as 17 localities with meadow and forest habitats in the Slovak part of the range (Figure 1). The investigations were undertaken during a series of research and monitoring projects with a large variety of methodological approaches. Here we only briefly summarize the four main methods used for the collection of isopods: 1) repeated individual sampling at favorable microsites; 2) pitfall trapping (different numbers of traps per locality, different time of exposition); 3) heat extraction of isopods from soil samples (usually 3–5 samples taken several times per year); 4) heat extraction of isopods from sieved litter sampled in selected (primarily forest) localities. The majority of the localities were intensively studied for one or two years. Most are under nature protection as National Nature Reserves (NNR), Nature Reserves (NR) and Nature Monuments (NM). Short descriptions of the sites are provided below and are distinguished into either Czech or Slovak subgroups. The localities are listed alphabetically. For more detailed characteristics, see Kuča et al. (1992), Mackovčin et al. (2002) and Jongepierová (2008).

Localities in the Czech part of the White Carpathians:

1 Bílé potoky NR – 49°06'56"N, 18°01'39"E, 380–500 m a.s.l., two meadow enclaves surrounded by mixed deciduous forests, 120 years old.

2 Brumov – 49°05'58"N, 18°01'59"E, 400 m a.s.l., meadow with traditional pasture management.

3 Čertoryje NNR – 48°51'31"N, 17°24'42"E, 350–445 m a.s.l., meadow (*Cirsio-Brachypodionpinnati*) with solitary oak and lime trees.

4 Chladný vrch NM – 49°01'31"N, 18°00'32"E, 550–575 m a.s.l., beech forest (*Caricipilosae*-*Fagetum*), 150–170 years old.

5 Drahy NR – 48°55'16"N, 17°38'16"E, 400–513 m a.s.l., meadow (*Cirsio*-*Brachypodionpinnati*).

6 Hrozenkovský lom – 48°58'24"N, 17°52'15"E, 500–520 m a.s.l., abandoned basalt quarry with mixture of grassland and forest vegetation.

7 Hutě NR – 48°59'26"N, 17°54'30"E, 450–535 m a.s.l., meadows and pastures (*Anthoxantho-Agrostietum*) with beech forest fragments.

8 Lopenické sedlo – 48°56'20"N, 17°48'00"E, 700 m a.s.l., pasture.

9 Javořina NNR – 48°51'34"N, 17°40'27"E, 835–970 m a.s.l., beech forest (*Dentarioenneaphylli*-*Fagetum*, *Lunario-Aceretum*).

10 Jazevčí NNR – 48°52'18"N, 17°33'45"E, 340–473 m a.s.l., meadow (*Cirsio-Brachypodionpinnati*) and pasture.

11 Okrouhlá NR – 49°02'48"N, 18°03'27"E, 620–655 m a.s.l., mixed beech forest (predominantly *Fagussylvatica*, as well as other deciduous tree species), 130 years old.

12 Ploščiny NR – 49°08'18"N, 18°03'40"E, 670–739 m a.s.l., meadow with dispersed trees (*Carpinusbetulus*, *Juniperuscommunis*, *Fagussylvatica*, *Abiesalba*).

13 Pod Hribovňou NM – 48°55'58"N, 17°50'43"E, 550–640 m a.s.l., meadows and pastures (*Anthoxantho-Agrostietum*) with solitary trees.

14 Pod Vrchy NM – 49°04'37"N, 17°56'21"E, 330–370 m a.s.l., hornbeam forest (*Caricipilosae-Carpetinum*), 70 years old.

15 Pod Žitkovským vrchem NR – 48°59'11"N, 17°52'59"E, 480–620 m a.s.l., meadows and pastures (*Violioncaninae*, *Calthion*) with forest fragments.

16 Porážky NNR – 48°53'08"N, 17°37'26"E, 540–610 m a.s.l., meadow (*Cirsio-Brachypodionpinnati, Angelico*-*Cirsietumoleracei*).

17 Sidonie NR – 49°03'09"N, 18°04'24"E, 425–560 m a.s.l., old and well-preserved beech forest with a predominance of *Fagussylvatica*, 170 years old.

18 Skaličí – 48°59'40"N, 17°52'53"E, 600–630 m a.s.l., limestone block outcrop with forest growth (*Fagussylvatica*).

19 Strání – 48°54'10"N, 17°40'55"E, 490–500 m a.s.l., intensively grazed pasture.

20 Trnovský mlýn – 48°53'47"N, 17°34'44"E, 450 m a.s.l., pasture.

21 Uvezené NM – 48°54'30"N, 17°38'53"E, 490–570 m a.s.l., hornbeam forest (*Caricipilosae*-*Carpinetum*).

22 U Zvonice NM – 48°56'23"N, 17°47'20"E, 630–670 m a.s.l., meadow (*Anthoxantho-Agrostietum*, *Filipendulenion*).

23 Vápenky NM – 48°52'31"N, 17°38'27"E, 470–570 m a.s.l., beech forest (*Caricipilosae-Fagetum*).

24 Ve Vlčí NR – 48°55'47"N, 17°51'24"E, 580–720 m a.s.l., pastures (*Anthoxantho*-*Agrostietum*) with forest fragments (*Fagussylvatica*).

25 Výzkum – 48°50'27"N, 17°33'25"E 400–425 m a.s.l., meadow, an experimental area for the monitoring of successional development of herbaceous-rich grasslands in the area.

26 Záhumenice NM – 48°53'42"N, 17°41'09"E, 500 m a.s.l., mosaic of meadow habitats (*Calthion*, *Sparganio*-*Glycerionfluitantis*, *Cirsio*-*Brachypodionpinnati*).

Localities in the Slovak part of the White Carpathians:

27 Babiná NM – 49°02'05"N, 18°10'40"E, 310–400 m a.s.l., xerothermic forest-steppe habitats on slopes with southwestern aspect.

28 Blažejová NM – 48°52'34"N, 17°49'07"E, 400–440 m a.s.l., typical meadows with orchids on western slopes with local springs.

29 Brezovská dolina NM – 49°05'28"N, 18°08'36"E, 440–470 m a.s.l., meadow locality with solitary trees, lime tufa and landslide springs.

30 Bučkova Jama NM – 48°49'07"N, 17°26'23"E, 480–550 m a.s.l., mosaic complex of preserved White Carpathian meadows and forests.

31 Chvojnica NM – 48°46'42"N, 17°22'42"E, 300–550 m a.s.l., narrow aluvium of the Chvojnica brook, in summer represented only by a set of puddles.

32 Fráterka – 48°49'55"N, 17°18'56"E, 375 m a.s.l., hornbeam forest (*Caricipilosae*-*Carpinetum*) near a hunting lodge of the same name at Skalica.

33 Grúň NM – 48°53'42"N, 17°47'56"E, 390–490 m a.s.l., mosaic of mesophilous and wet meadows with solitary trees.

34 Kožíkov vrch NM – 48°46'11"N, 17°29'21"E, 390–420 m a.s.l., old abandoned field, currently a mowed meadow.

35 Krivoklátska Tiesňava NM – 49°02'53"N, 18°09'05"E, 350–450 m a.s.l., limestone outcrops with beech and mixed forest growth.

36 Kurinov vrch NM – 48°52'43"N, 17°53'26"E, 425 m a.s.l., meadows on tufa terraces with characteristic vegetation, surrounded by forests.

37 Malejov NM – 48°46'19"N, 17°28'36"E, 420–430 m a.s.l., fragments of White Carpathian wet and dry meadows.

38 Mravcové NM – 48°54'26"N, 17°45'53"E, 475–500 m a.s.l., wet meadows with tufa and solitary trees.

39 Nebrová NR – 49°07'03"N, 18°07'27"E, 500–520 m a.s.l., alluvial alder growth (*Alnetum*) along small brooks.

40 Šmatlavé Uhlisko NR – 48°50'29"N, 17°19'14"E, 400 m a.s.l., hornbeam forest (*Caricipilosae*-*Carpinetum*)

41 Štefanová NM – 48°49'44"N, 17°26'44"E, 520–560 m a.s.l., herbaceous-rich meadows irregularly mowed.

42 Veľká Javorina NR – 48°51'39"N, 17°41'37"E, 860–870 m a.s.l., beech and maple forests (*Acero*-*Fagetum*, *Acero*-*Fagetum Lunarietosum*, *Fagetumpauper*) on the southeastern slopes.

43 Zábava – 49°01'35"N, 18°04'11"E, 280 m a.s.l., riparian vegetation along the Vlára Brook near Zábava-Horné Srnie village.

Given that the data were attained in different years using different methods, it was not possible to compare all parameters of isopod assemblages in detail. Therefore, this paper presents a general overview of the fauna of terrestrial isopods in the study area. For analysis of isopod assemblages according to their presence or absence, the programme CANOCO 5, unconstrained analysis, DCA (Lepš and Šmilauer 2014) was used.

## Results

In total, 19 species of terrestrial isopods belonging to nine families (see Appendix 1) were recorded for the whole area of the White Carpathians. For the region belonging to the Czech Republic, 16 species were found (Table 1), and in the Slovak region, 14 species were found (Table 2). Assemblages of isopods at individual localities consisted of one to ten species. The species with the highest frequency of occurrence within the whole study area was *Protracheoniscuspolitus* (22 and 12 localities in the Czech and Slovakian parts, respectively), *Trachelipusrathkii* (22 and 8 localities), *Ligidiumhypnorum* (19 and 12 localities) and *Porcelliumcollicola* (13 and 10 localities). These species appear to be typical of the White Carpathians. Two species categorized as relic species (i.e., species that exclusively inhabit undisturbed, nature closest habitats with low level of human impact, cf. Tuf and Tufová 2008) were recorded: the Carpathian endemic *Hyloniscusmariae* at the locality Javořina, and *Ligidiumgermanicum* in eleven forest localities. In the Czech part, the community with the highest degree of species richness was found at the locality Pod Hribovňou (locality 13 with 10 species), containing a mozaic of meadows, pastures and solitary trees. In addition, other species-rich communities, with nine isopod species, were recorded in the localities with a mosaic vegetation structure, Pod Žitkovským vrchem (locality 15) and Čertoryje (locality 3), or in the well-preserved beech forest of the Javořina (locality 9). In the Slovak part of the territory, the highest species richness with ten species was recorded in the meadow restored from an abandoned field at Kožíkov vrch (locality 34). High number of species was also found in the narrow alluvium of the Chvojnica brook and in the mozaic of mesophilous and wet meadows at Grúň (localities 31 and 33, both with eight species). Evidently, in the study area, well-preserved natural habitats support a relatively rich isopod fauna.

**Table 1.** Terrestrial isopods recorded in individual localities of the White Carpathians, in the Czech Republic, their presence (+), absence (–) and frequency of occurrence (F%). For numbers and description of localities, see Materials and methods.

Localities	1	2	3	4	5	6	7	8	9	10	11	12	13	14	15	16	17	18	19	20	21	22	23	24	25	26	Total	F(%)
* Ligidium germanicum *	–	–	–	+	–	–	+	–	+	–	+	–	+	+	+	+	+	+	–	–	–	–	–	+	–	–	11	42.3
* Ligidium hypnorum *	–	–	+	+	–	+	+	–	+	+	+	+	+	+	+	+	+	+	–	–	+	+	+	+	+	–	19	73.1
* Haplophthalmus mengii *	–	–	+	–	–	–	–	–	–	–	–	–	–	–	–	–	–	–	–	–	–	–	+	–	–	–	2	7.7
* Hyloniscus mariae *	–	–	–	–	–	–	–	–	+	–	–	–	–	–	–	–	–	–	–	–	–	–	–	–	–	–	1	3.8
* Hyloniscus riparius *	–	–	+	–	+	–	+	–	+	–	–	–	+	+	+	–	–	+	–	–	+	–	+	+	+	–	12	46.2
* Trichoniscus pusillus *	+	–	+	–	–	–	–	–	+	–	–	–	+	+	+	–	+	–	–	–	+	–	+	+	+	–	11	42.3
* Platyarthrus hoffmannseggii *	–	–	+	–	+	–	–	–	–	+	–	–	–	–	–	–	–	–	–	–	–	–	–	–	+	–	4	15.4
* Lepidoniscus minutus *	–	–	–	+	–	+	–	–	–	–	+	–	+	+	–	+	+	+	–	–	–	–	–	–	–	–	8	30.8
* Cylisticus convexus *	–	–	–	–	–	+	–	–	–	–	–	–	–	–	–	–	–	–	–	–	–	–	–	–	–	–	1	3.8
* Orthometopon planum *	–	–	–	–	–	–	–	–	–	–	–	–	–	–	–	–	–	–	–	–	–	–	–	–	–	–	0	–
* Protracheoniscus politus *	+	–	+	+	+	+	+	–	+	–	+	+	+	+	+	+	+	+	+	–	+	+	+	+	+	+	22	84.6
* Porcellium collicola *	–	+	+	–	+	+	+	+	–	+	–	–	+	–	+	–	–	–	–	+	+	+	–	+	–	–	13	50.0
* Porcellium conspersum *	–	–	–	–	–	–	–	–	+	–	–	–	+	–	+	+	–	–	–	–	–	–	–	–	–	–	4	15.4
* Trachelipus nodulosus *	–	–	–	–	–	–	–	–	–	–	–	–	–	–	–	–	–	–	–	–	–	–	–	–	–	–	0	–
* Trachelipus rathkii *	+	+	+	+	+	–	+	–	+	+	+	+	+	+	+	+	+	+	+	–	–	+	+	+	+	+	22	84.6
* Trachelipus ratzeburgii *	+	–	–	+	–	–	+	–	+	–	+	+	+	+	+	–	+	+	–	–	+	–	–	+	–	+	14	53.8
* Porcellio scaber *	–	–	–	–	–	–	–	–	–	–	–	–	–	–	–	–	–	–	–	–	–	–	–	–	–	–	0	–
* Porcellionides pruinosus *	–	–	–	–	+	–	–	–	–	–	–	–	–	–	–	–	–	–	–	–	–	–	–	–	+	–	2	7.7
* Armadillidium vulgare *	–	+	+	–	+	–	–	–	–	+	–	–	–	–	–	+	–	–	+	+	–	–	+	–	+	+	10	38.5
Total number of species	4	3	9	6	7	5	7	1	9	5	6	4	10	8	9	7	7	7	3	2	6	4	7	8	8	4		

**Table 2.** Terrestrial isopods recorded in individual localities of the White Carpathians, in Slovakia, with their presence (+), absence (–), and frequency of occurrence (F%). Numbers of localities, see Materials and methods.

Localities		27	28	29	30	31	32	33	34	35	36	37	38	39	40	41	42	43	Total	F(%)
* Ligidium germanicum *	*LigdHypn*	–	–	+	–	–	–	+	–	–	–	–	+	–	–	–	–	–	3	17.6
* Ligidium hypnorum *	*LigdGerm*	–	+	+	+	+	–	+	+	–	–	+	+	+	–	+	+	+	12	70.6
* Haplophthalmus mengii *	*HaplMeng*	–	–	–	–	–	–	–	–	–	–	–	–	–	–	–	–	–	0	–
* Hyloniscus mariae *	*HylnMari*	–	–	–	–	–	–	–	–	–	–	–	–	–	–	–	–	–	0	–
* Hyloniscus riparius *	*HylnRipr*	–	–	–	+	+	–	+	+	–	–	+	–	–	–	–	–	–	5	29.4
* Trichoniscus pusillus *	*TricPuls*	–	–	–	–	+	–	+	+	–	–	–	–	–	+	+	–	+	6	35.3
* Platyarthrus hoffmannseggii *	*PlatHoff*	–	–	–	–	–	–	–	–	–	–	–	–	–	–	–	–	–	0	–
* Lepidoniscus minutus *	*LepdMint*	–	–	–	–	–	–	–	–	–	–	–	–	–	–	–	–	–	0	–
* Cylisticus convexus *	*CylsConv*	+	–	–	–	–	–	–	–	–	–	–	–	–	–	–	–	–	1	5.9
* Orthometopon planum *	*OrthPlan*	–	+	–	–	–	–	–	–	–	–	–	–	–	–	–	–	–	1	5.9
* Protracheoniscus politus *	*ProtPolt*	–	+	+	+	+	+	+	+	+	–	–	+	–	+	+	+	–	12	70.6
* Porcellium collicola *	*PorcColl*	–	+	+	+	+	–	+	+	+	–	+	+	–	–	+	–	–	10	58.8
* Porcellium conspersum *	*PorcCons*	–	–	–	–	–	–	–	–	–	–	–	–	–	–	–	–	–	0	–
* Trachelipus nodulosus *	*TracNodl*	+	–	–	–	–	–	–	–	–	+	–	–	–	–	–	–	–	2	11.8
* Trachelipus rathkii *	*TracRath*	–	+	–	+	+	–	+	+	–	–	+	+	–	–	+	–	–	8	47.1
* Trachelipus ratzeburgii *	*TracRatz*	–	–	–	–	+	–	–	+	–	–	+	–	–	–	–	–	–	3	17.6
* Porcellio scaber *	*PorcScab*	–	–	–	–	–	–	–	+	–	–	–	–	–	–	–	–	–	1	5.9
* Porcellionides pruinosus *	*PorcPrui*	–	+	–	–	–	–	+	+	–	–	–	–	–	–	–	–	–	3	17.6
* Armadillidium vulgare *	*ArmdVulg*	–	–	–	+	+	–	–	+	–	–	+	+	–	–	+	–	+	7	41.2
Number of species		2	6	4	6	8	1	8	10	2	1	6	6	1	2	6	2	3		

### Similarity of assemblages of the White Carpathians

**Figure 2.**
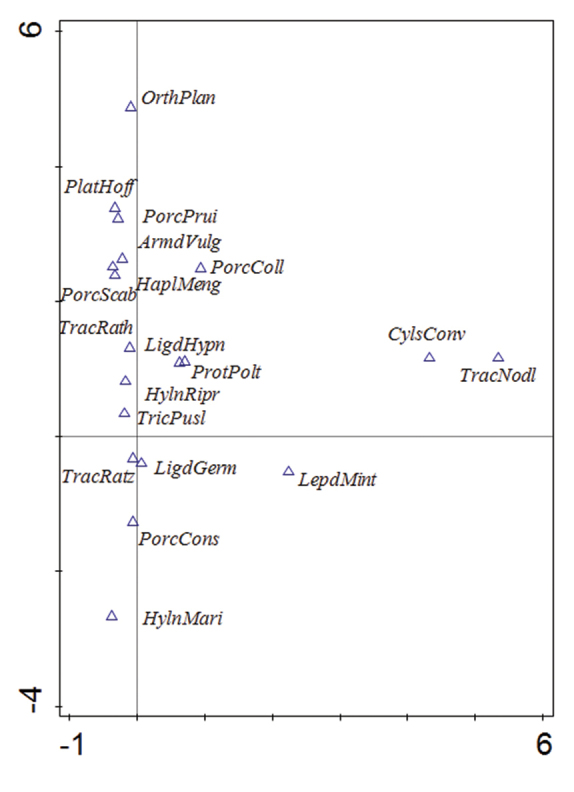
The ordination analysis of isopod species recorded at individual study localities in the White Carpathians (CANOCO 5, unconstrained analysis, DCA). For abbreviation of species’ names, see Table 2.

Given that the analyzed data came from different studies, we compared the assemblages of terrestrial isopods at individual localities according to the presence or absence of the species only. Frequently occurring species were clustered in the first and second quadrat, primarily along the y-axis (Figure 2). A more isolated position was typical of relatively rare species with a small number of records, such as *Orthometoponplanum*, *Cylisticusconvexus*, *Trachelipusnodulosus*, and *H.mariae*.

**Figure 3.**
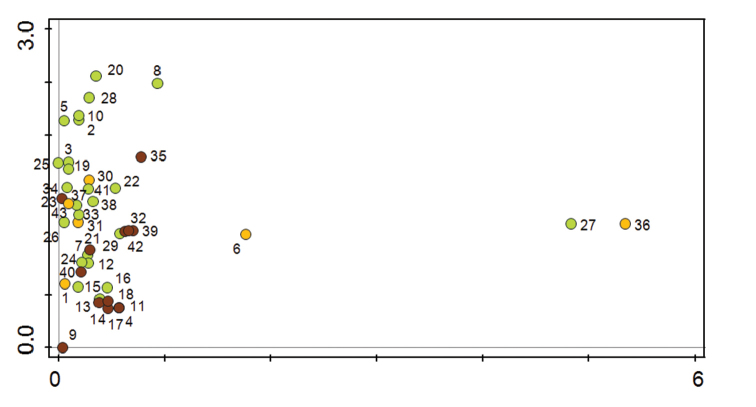
The ordination analysis (CANOCO 5, unconstrained analysis, DCA) of individual localities (1–43) in the White Carpathians according to present terrestrial isopod assemblages. For numbers of individual localities, see Materials and methods. Key: brown spots, forest localities; light green spots, meadows and pastures; yellow spots, localities of mixed meadows and woods.

The dense clustering of localities (Figure 3) corresponds with relatively high rates of similarity of isopod assemblages in most of the studied meadow and forest sites. Nevertheless, a certain gradient from meadow to forest localities can be distingushed. Isopod assemblages in herbaceous-rich natural meadow localities (numbers 2, 3, 5, 8, 10, 19, 20, 25, 28) are isolated and situated in the upper part of the biplot. Their position corresponds with the species *O.planum*, *Platyarthrushoffmannseggii* (obtained only by soil sampling) and *Porcellionidespriunosus* or *Armadillidiumvulgare* (Figure 2). Further down (closer to the intersection of the axes) are clustered many sites of forest and non-forest character; forest localities are generally situated in the lower half of the whole biplot as a whole. The analysis separated out several localities, which probably represent most xerothermic and open habitats. Locality 6, Hrozenkovský lom quarry, was partly separated to the right of the others owing to the presence of *Cylisticusconvexus*. A distinct separation of two localities, xerothermic forest-steppe habitats at Babinná (locality 27) and meadow on tufa terraces at Kurinov vrch (locality 36), corresponds only with the finding of individual species of *T.nodulosus* (at both localities) and *C.convexus* (locality 27).

## Discussion

A total of 43 species of terrestrial isopods are currently known in the Czech Republic, hence our material pertaining to the Czech part of the White Carpathians represents 37 % of Czech fauna. Similarly, in the Slovak part of the White Carpathians, the 14 recorded species represent approximately 31 % of total known Slovak fauna (45 species). Given that in half of the localities, isopod communities were composed of six to 10 species, we can consider the White Carpathians rich in woodlice fauna. The data from localities with only three or fewer species should be considered an underestimation due to the sampling method and effort. Additional surveys would certainly increase total numbers through other frequently occurring species.

It must be mentioned that our study summarizes data only from natural and not synanthropic habitats. In comparison with other areas heretofore explored in the Western Carpathians, this represents another rich area after the Little Carpathians (30 species, Flasar and Flasarová 1989, Flasarová 1980, 1986), Bükk Mts (24 species, Allspach 1996, Forró and Farkas 1998, Kontschán 2004), Aggtelek and Slovak Karst (both 20 species, Forró and Farkas 1998, Kontschán 2004, Vilisics et al. 2008, Frankenberger 1940, Flasarová 1994, 1998) and Pieniny (19 species, Hudáková and Mock 2006). The Little Carpathians constitute the neighboring area, and so we can expect more species to be present in the White Carpathians. Thus the White Carpathians, especially their Slovak part, deserve further attention. However, it should be acknowledged that the large number of species in the Little Carpathians can also be linked to the fact that Flasarová (1986) collected material both in natural and anthropogenic habitats, whereas in all other species-rich regions isopods were collected in more or less natural biotopes. The urban environment offers higher microhabitat diversity and favorable conditions for synanthropic species, illustrated by the fact that more species-rich communities can be found in the cities (Riedel et al. 2009). Given that the access to calcium represents an important factor influencing the distribution of terrestrial isopods (Sutton 1972), karstic regions are richer in species than others (Vilisics et al. 2008). In forest habitats, the number of species at one locality usually varies from three to seven (Farkas et al. 1999, Tajovský 2002), hence the forest localities of the White Carpathians with species ranges of six to 10 are very rich in isopod fauna.

In the present study, we surveyed a relatively wide spectrum of biotopes in the White Carpathians. In total, we sampled a range of forest, meadow and pasture sites as well as sites with a mixture of habitats. Differences in isopod species composition were observed, including between forest biotopes, cultivated sites and pastures (e.g., Paoletti 1987). However, some species in Central Europe are eurytopic, very common and widely distributed. For example, *Trachelipusrathkii* is typical of disturbed and open habitats in the initial phases of succession on colliery heaps (Tajovský 2001), as well as being common in wetlands and the floodplain forests in Hungary (Farkas 1998) and the Czech Republic (Tajovský 1998), yet in other areas it can avoid forests (Schmidt 1997). In our study area, it was present in nearly all forest sites. It has been acknowledged that other species typical of forests (*Lepidoniscusminutus*, *Trachelipusratzeburgii*, *Protracheoniscuspolitus*) rarely penetrate open habitats. This statement was confirmed in our study, with the exception of *P.politus*, which was found in almost all localities. Typical inhabitants of the White Carpathian meadows and pastures include *Armadillidiumvulgare* and *T.rathkii*. Both are ubiquitous and able to colonise forest habitats. *Armadillidiumvulgare*, a species introduced in numerous parts of the world by human activities, is often connected with stony habitats (Schmalfuss 2003) and is viewed as less common in forest stands (Allspach 1996). Given that it was only present in one forest locality, Vápenky (protected as a Nature Monument, albeit somewhat influenced by human activities), we consider the White Carpathian forest localities more or less undisturbed and thus of high conservation value. Species with high levels of affinity to woodlands with moist and shady sites (*Hyloniscusriparius*, *Trichoniscuspusillus*) can also be found in grasslands (Sutton 1968). Their occurrence in the meadows and pastures studied corresponds with wet patches typical of some White Carpathian grasslands (Mackovčin et al. 2002). We can conclude that despite being open ecosystems the meadows in highland areas support hygrophilous and forest species similar to those in forest sites (Tomescu et al. 2005).

From a zoogeographical point of view, European and Central European species predominated (Schmalfuss 2003). *Lepidoniscusminutus*, *P.politus*, *H.riparius*, *Porcelliumcollicola*, and *Ligidiumgermanicum* form a group that is distributed from Central Europe to the Balkan Peninsula. Their common occurrence may be explained by the fact that following the last glaciation, a significant proportion of contemporary Central European fauna migrated from the Balkans to the Carpathian Basin (Farkas 2007).

The meadows and pastures of the White Carpathians have in fact been formed and influenced by humans for numerous centuries (cf. Mackovčin et al. 2002). Nevertheless, a lack of introduced and synanthropic species reveals a weak influence on present-day isopod fauna. Only one species, *Porcellionidespruinosus*, which can be considered introduced, was recorded. Nevertheless, its presence is faunistically interesting because the White Carpathians appear to represent the northernmost limit of its apparently original South European or Mediterranean distribution. Further north, this species is known only in synanthropic sites (Frankenberger 1959). The presence of the synathropic species *Porcellioscaber* at the locality Kožíkov vrch can be related to this habitat, representing an old, abandoned field that had been transformed into a regularly mown meadow.

The record of the Carpathian endemic *Hyloniscusmariae* is very important. It was found at the Javořina National Nature Reserve, a locality with great biodiversity, predominatly including old and partly krummholz-like beech forest at the peak of the highest mountain. According to the current Red List of Threatened Species in the Czech Republic (Tajovský and Tuf 2017), this species is categorized as endangered. The presence of *Orthometoponplanum* and *Trachelipusnodulosus* confirmed the spread and penetration of termophilous or xerothermic species to this area from the South.

When evaluating the (dis)similarity of communities of the White Carpathians, a northeast-southwest geographical as well as ecological gradient (meadow – pasture – forest) was observed. The analysis divided the localities into herbaceous-rich meadow sites and other meadows and forests with relatively rich isopod fauna, and distinguished several specific (and mostly xerothermic) sites (Figs 2 and 3). Indeed, the White Carpathians were deemed a valuable area due to their considerable biodiversity (Webster et al. 2001).

In conclusion, the recorded number of species, their distribution within meadows, pastures and forests, the occurrence of species-rich communities (especially in forest habitats), and the presence of the relic species, *Hyloniscusmariae* and *Ligidiumgermanicum*, together with the absence of introduced and ubiquitous species, indicate the high nature conservancy value of the whole area. The diversity of habitats in the White Carpathians presents a favorable environment for rich communities of terrestrial isopods in the Central European region.

## Appendix 1

**Systematic list of the species of terrestrial isopods recorded in the White Carpathians, the Czech Republic, and Slovakia**

**Suborder Oniscidea**

Family Ligiidae

*Ligidiumgermanicum* Verhoeff, 1901

*Ligidiumhypnorum* (Cuvier, 1792)

Family Trichoniscidae

*Haplophthalmusmengii* (Zaddach, 1844)

*Hyloniscusmariae* Verhoeff, 1908

*Hyloniscusriparius* (C. Koch, 1838)

*Trichoniscuspusillus* Brandt, 1833

Family Platyarthridae

*Platyarthrushoffmannseggii* Brandt, 1833

Family Philosciidae

*Lepidoniscusminutus* (C. Koch, 1838)

Family Cylisticidae

*Cylisticusconvexus* (De Geer, 1778)

Family Agnaridae

*Orthometoponplanum* (Budde-Lund, 1885)

*Protracheoniscuspolitus* (C. Koch, 1841)

Family Trachelipodidae

*Porcelliumcollicola* (Verhoeff, 1907)

*Porcelliumconspersum* (C. Koch, 1841)

*Trachelipusnodulosus* (C. Koch, 1838)

*Trachelipusrathkii* (Brandt, 1833)

*Trachelipusratzeburgii* (Brandt, 1833)

Family Porcellionidae

*Porcellioscaber* Latreille, 1804

*Porcellionidespruinosus* (Brandt, 1833)

Family Armadillidiidae

*Armadillidiumvulgare* (Latreille, 1804)

## References

[B1] Allspach A (1996) The terrestrial isopods of the Bükk National Park (Crustacea; Isopoda; Oniscidea). In: Mahunka S (Ed.) The fauna of the Bükk National Park, II.Hungarian Natural History Museum, Budapest, 71–74.

[B2] Farkas S (1998) Population dynamics, spatial distribution, and sex ratio of *Trachelipusrathkei* Brandt (Isopoda: Oniscidea) in a wetland forest by the Drava River. Israel Journal of Zoology 44(3–4): 323–331.

[B3] Farkas S (2007) The terrestrial isopod fauna of South Transdanubia (Hungary). Somogyi Múzeumok Közleményei 17: 159–168.

[B4] Farkas S Hornung E Morschhause T (1999) Composition of isopod assemblages in different habitat types. In: Tajovský K Pižl V (Eds) Soil Zoology in Central Europe.Institute of Soil Biology AS CR, České Budějovice, April 1999, 37–44.

[B5] Flasar I Flasarová M (1989) Ergänzungen zur Monographie ”The soil fauna of the Little Carpathians“ (Mollusca et Isopoda). Faunistische Abhandlůungen Museum Tierkunde Dresden 17: 1–18.

[B6] Flasarová M (1958) K poznání moravskoslezských Oniscoideí. Časopis Slezského muzea v Opavě (A) 7: 100–130.

[B7] Flasarová M (1980) *Hyloniscustranssilvanicus* (Verhoeff, 1901) im Gebirge Malé Karpaty in der Westslowakei. Faunistische Abhandlungen, Staatliches Museum für Tierkunde Dresden 7: 273–278.

[B8] Flasarová M (1986) Isopoda (Asellota, Oniscidea) of the Little Carpathians. In: Nosek J (Ed.) The Soil Fauna of the Little Carpathians.Slovak Academy of Science, Bratislava, 183–216.

[B9] Flasarová M (1994) Über einige Landasseln aus der Slowakei, gesammelt von Herrn Dr. Ján Brtek (Crustacea: Isopoda: Oniscidea). Faunistische Abhandlungen Staatliches Museum für Tierkunde Dresden 19: 135–140.

[B10] Flasarová M (1998) Bericht über Isopoden (Asellota et Oniscidea) im Slowakischen Donaugebiet. Acta Musei Nationalis Pragae, Series B, Historia Naturalis 54: 61–78.

[B11] Forró L Farkas S (1998) Checklist, preliminary distribution maps and bibliography of woodlice in Hungary (Isopoda: Oniscidea). Miscellanea Zoologica Hungarica 12: 21–44.

[B12] Frankenberger Z (1940) Oniscoidea Slovakiae. Sborník entomologického Oddělení Národního Musea v Praze 18: 60–69.

[B13] Frankenberger Z (1941) Poznámky o českých Isopodech. II. Věda přírodní, Praha 20(5): 151–152.

[B14] Frankenberger Z (1942) Poznámky o českých Isopodech. III. Věda přírodní, Praha 21(3): 85–88; (4): 119–121.

[B15] Frankenberger Z (1944) Oniscoidea Čech a Moravy. Věstník Královské české společnosti nauk, Praha 1944(8): 1–28.

[B16] Frankenberger Z (1954) Řád: Stejnonožci – Isopoda. In: Hrabě S (Ed.) Klíč zvířeny ČSR, 1.NČSAV, Praha, 499–507.

[B17] Frankenberger Z (1959) Stejnonožci suchozemští (Oniscoidea). Fauna ČSR, 14. NČSAV, Praha, 212 pp.

[B18] Hopkin SP Hardisty GN Martin MH (1986) The woodlouse *Porcellioscaber* as a ‘biological indicator’ of Zinc, Cadmium, Lead and Copper pollution. Environmental Pollution (Series B) 11: 271–290. 10.1016/0143-148X(86)90045-5

[B19] Hudáková J Mock A (2006) Suchozemské rovnakonôžky (Isopoda: Oniscidea) Pieninského národného parku. Entomofauna carpathica 18: 47–55.

[B20] Jongepierová I (Ed.) (2008) Louky Bílých Karpat (Grasslands of the White Carpathian Mountains). ZO ČSOP Bílé Karpaty, Veselí nad Moravou, 461 pp.

[B21] Kontschán J (2004) Néhány adat az Északi-középhegység ászkarák faunájához (Crustacea: Isopoda: Oniscidea). Folia Musei Historico Naturalis Matrensis 28: 91–93.

[B22] Kuracina D Kabátová A (2005) Terestrické rovnakonôžky (Crustacea: Isopoda: Oniscoidea) In: Majzlan O (Ed.) Fauna Devínskej Kobyly.APOP, Bratislava, 56–57.

[B23] Lepš J Šmilauer P (2014) Multivariate analysis of ecological data using CANOCO 5. Cambridge University Press, Cambridge, 362 pp.

[B24] Mackovčin P Jatiová M et al. (2002) Zlínsko. Chráněná území ČR, Volume II. Agentura ochrany přírody a krajiny ČR a EkoCentrum Brno, Praha, 376 pp.

[B25] Paoletti MG (1987) Terrestrial isopods in agroecosystems of the low-lying plain in north-eastern Italy. In: Striganova BR (Ed.) Soil Fauna and Soil Fertility.Proceedings of the 9^th^ International Colloquium on Soil Zoology, Moscow, August 1985. Nauka, Moscow, 423–426.

[B26] Paoletti MG Hassall M (1999) Woodlice (Isopoda: Oniscidea): their potential for assessing sustainability and use as bioindicators. Agriculture, Ecosystems & Environment 74: 157–165. 10.1016/S0167-8809(99)00035-3

[B27] Riedel P Navrátil M Tuf IH Tufová J (2009) Terrestrial isopods (Isopoda: Oniscidea) and millipedes (Diplopoda) of the City of Olomouc. In: Tajovský K Schlaghamerský J Pižl V (Eds) Contributions to Soil Zoology in Central Europe III.Institute of Soil Biology, Biology Centre, AS CR, v.v.i., České Budějovice, 125–132.

[B28] Schmalfuss H (2003) World catalog of terrestrial isopods (Isopoda: Oniscidea). Stuttgarter Beiträge zur Naturkunde, Serie A 654: 1–341.

[B29] Schmidt C (1997) Revision of the European species of the genus *Trachelipus* Budde-Lund, 1908 (Crustacea: Isopoda: Oniscidea). Zoolgical Journal of the Linnean Society 121: 129–244. 10.1111/j.1096-3642.1997.tb00337.x

[B30] Spitzer L Tuf IH Tufová J Tropek R (2007) Příspěvek k poznání fauny epigeických bezobratlých dvou přírodních jedlobukových lesů ve Vsetínských vrších (Česká republika). Práce a Studie Muzea Beskyd (Přírodní vědy) 19: 71–82.

[B31] Sutton SL (1968) The population dynamics of *Trichoniscuspusillus* and *Philosciamuscorum* (Crustacea, Oniscoidea) in limestone grassland. Journal of Animal Ecology 37: 425–444. 10.2307/2958

[B32] Sutton SL (1972) Invertebrate types – Woodlice. Ginn & Company limited, London, 153 pp.

[B33] Štrichelová J Tuf IH (2012) Terrestrial isopods (Crustacea: Isopoda, Oniscidea). In: Holecová M Christophoryová J Mrva M Roháčová M Stašiov S Štrichelová J Šustek Z Tirjaková E Tuf IH Vďačný P Zlinská J (Eds) Biodiversity of soil micro- and macrofauna in oak-hornbeam forest ecosystem on the territory of Bratislava.Comenius University in Bratislava, Bratislava, 43–48.

[B34] Tajovský K (1998) Diversity of terrestrial isopods (Oniscidea) in flooded and nonflooded ecosystems of southern Moravia, Czech Republic. Israel Journal of Zoology 44(3–4): 311–322.

[B35] Tajovský K (2001) Colonization of colliery spoil heaps by millipedes (Diplopoda) and terrestrial isopods (Oniscidea) in the Sokolov region, Czech Republic. Restoration Ecology 9: 365–369. 10.1046/j.1526-100X.2001.94005.x

[B36] Tajovský K (2002) Mnohonožky (Diplopoda), stonožky (Chilopoda) a suchozemští stejnonožci (Oniscidea) Národní přírodní rezervace Žofínský prales v Novohradských horách. In: Papáček M (Ed.) Biodiverzita a přírodní podmínky Novohradských hor.JčU a EntÚ AV ČR, České Budějovice, 157–161.

[B37] Tajovský K (2008) Suchozemští stejnonožci (Oniscidea). In: Jongepierová I (Ed.) Louky Bílých Karpat (Grasslands of the White Carpathian Mountains).ZO ČSOP Bílé Karpaty, Veselí nad Moravou, 217–218.

[B38] Tajovský K Tuf IH (2017) Oniscidea (suchozemští stejnonožci). In: Hejda R Farkač J Chobot K (Eds) Červený seznam ohrožených druhů České republiky, Bezobratlí (Red List of threatened species of the Czech Republic, Invertebrates).Příroda 36: 105–107.

[B39] Tomescu N Muresan D Olaru L Hotea R (2005) Terrestrial isopod communities (Crustacea, Isopoda) in riverside coppices and meadows of mountainous hilly and depression areas. Studia Universitatis Babes-Bolyai, Biologia 50(2): 19–25.

[B40] Tuf IH Tufová J (2005) Communities of terrestrial isopods (Crustacea: Isopoda: Oniscidea) in epigeon of oak-hornbeam forests of SW Slovakia. Ekológia (Bratislava) 24 Supplement 2/2005: 113–123.

[B41] Tuf IH Tufová J (2008) Proposal of ecological classification of centipede, millipede and terrestrial isopod faunas for evaluation of habitat quality in Czech Republic. Časopis Slezského Muzea Opava (A) 57: 37–44.

[B42] Tufová J Tuf IH (2003) Druhové bohatství půdních bezobratlých - metodologický artefakt. In: Štykar J (Ed.) Geobiocenologie a její využití v péči o les a chráněná území.MZLU Brno & Školský lesní podnik Masarykův les Křtiny, Geobiocenologické spisy, svazek č. 7, 107–114.

[B43] Vilisics F Nagy A Sólymos P Farkas R Kemencei Z Páll-Gergely B Kisfali M Hornung E (2008) Data on the terrestrial isopod fauna of the Alsó-Hegy, Aggtelek National Park, Hungary. Folia faunistica Slovaca 13: 19–22.

[B44] Webster R Holt S Avis Ch (2001) The status of the Carpathians. A report developed as a part of The Carpathian Ecoregion Initiative. WWF. http://assets.panda.org/downloads/ceri_statusofthecarpathians_wwfdcp2001.pdf

